# Flow Sorting and Molecular Cytogenetic Identification of Individual Chromosomes of *Dasypyrum villosum L*. (*H. villosa*) by a Single DNA Probe

**DOI:** 10.1371/journal.pone.0050151

**Published:** 2012-11-20

**Authors:** Valentina Grosso, Anna Farina, Andrea Gennaro, Debora Giorgi, Sergio Lucretti

**Affiliations:** 1 ENEA, CASACCIA Research Center, Rome, Italy; 2 Department of Agriculture, Forestry, Nature and Energy - DAFNE, University of Tuscia, Viterbo, Italy; University of Connecticut, United States of America

## Abstract

*Dasypyrum villosum* (L.) *Candargy* (sin. *Haynaldia villosa*) is an annual wild diploid grass species (2n = 2x = 14; genome VV) belonging to the *Poaceae* family, which is considered to be an important source of biotic and abiotic stress resistance genes for wheat breeding. Enhanced characterization of *D. villosum* chromosomes can facilitate exploitation of its gene pool and its use in wheat breeding programs. Here we present the cytogenetic identification of *D. villosum* chromosomes on slide by fluorescent *in situ* hybridization (FISH), with the GAA simple sequence repeat (SSR) as a probe. We also describe the isolation and the flow cytometric analysis of *D. villosum* chromosomes in suspension, resulting in a distinguished flow karyotype. Chromosomes were flow sorted into three fractions, according their DNA content, one of which was composed of a single type of chromosome, namely 6 V, sorted with over 85% purity. Chromosome 6 V is known to carry genes to code for important resistance and seed storage characteristics, and its isolation represents a new source of genetic traits and specific markers useful for wheat improvement.

## Introduction

Cultivation, domestication and breeding in the elite crops over the past 10,000 years has greatly reduced their genetic variability [1], making them more vulnerable to diseases and pests and less adapted to stresses arising from environmental conditions and climate change [2]. Wild relatives and related species are important sources of genetic variability for cultivated plants, and one of the strategies of crop breeding is to incorporate favourable alleles by means of interspecific hybridization using classical breeding [3] and/or biotechnology techniques [4], [5], [6].

*Dasypyrum villosum* (L.) *Candargy* (*Dv*) (sin. *Haynaldia villosa*) is an annual diploid (2n = 2x = 14, genome VV) cross pollinating grass species distributed throughout the Mediterranean Region and is considered a valuable source of useful genes for wheat breeding [7], [8]. In fact, *D. villosum* has many useful agronomic traits, all reviewed by Gradzielewska [8], such as salt and drought tolerance, resistance to several wheat pathogens and pests, e.g. powdery mildew (*Erysiphe graminis* f. ssp. *tritici*), loose smuts (*Ustilago nuda* and *U. triticii*), eyespot (*Pseudocercosporella herpotricoides*, *Tapesia yallundae* and *T. acuformis*), stem and leaf rusts (*Puccinia gramnis* and *P. recondita*), *Septoria tritici*, wheat curl mite (*Aceria tosichella*) and wheat spindle streak mosaic virus (WSSMV). It also brings genetic loci controlling seed storage proteins that affect the amount, yield and quality of seed proteins [8]. Over the years, several attempts have been made to transfer genes from *D. villosum* into wheat through the development of chromosome addition, substitution and translocation lines [8], [9], [10], [11], [12], [13]. As it is usually the case with alien introgressions, alien fragments often carry large blocks of completely linked genes, some of which may have a negative impact on the recipient elite cultivar [14]. The way to resolve this problem is to reduce the size of the alien segment by repeated cycles of induced homoeologous recombination, followed by backcrossing. These steps require precise monitoring by cytogenetic analyses and/or by the use of DNA markers and prompts for additional cytogenetic studies on *D. villosum*. The first fluorescence *in situ* hybridization (FISH) characterization of the V genome was performed with DNA repetitive sequences as probes [15], but only four of the seven chromosomes of the haploid complement could be identified using a combination of four different DNA probes. Later, Yuan and Tomita [16], isolated the repetitive sequence pDvTU383, which, in combination with probes pTa71 [17] and pTa794 [18] consisting of 18S-5.8S-28S rDNA and 5S rDNA multigene families, respectively, permitted the identification of each of the seven pairs of *D. villosum* chromosomes. This approach still required the use of three different probes, thus making cytogenetic studies in this species rather complicated. Further use of *D. villosum* in wheat breeding programmes will necessitate the use of high-density genetic maps, but very few DNA markers are available for this wild grain [19], [20], [21]. An elegant way of saturating genetic maps at regions of interest is to develop chromosome-specific DNA markers from flow-sorted chromosomes [22]. Very pure fractions of individual chromosomes can be isolated in several species by flow sorting, the so-called *flow cytogenomic* (FC) approach [23]. This approach relies on high-quality chromosome suspensions and sufficient differences in the individual chromosome DNA content to produce a distinct flow karyotype. To date, flow cytometry analysis and sorting have been reported for only a few wild relatives of wheat [24]. To expand the range of species accessible via this approach, we have developed a procedure for the generation of chromosome suspensions, and we have evaluated the potential of flow cytometry for chromosome sorting in *D. villosum*. Here we give the first complete GAA ideogram of the whole *D. villosum* complement describing a model approach to the cytological identification of *D. villosum* chromosome by FISH using a microsatellite probe and minor chromosome morphological variations, and we reveal the first *D. villosum* flow karyotype. Three regions were identified for flow sorting, and the chromosome content of each has been FISH characterized, demonstrating the isolation of a high-purity 6 V single chromosome fraction. Our results will make it possible to use the FC approach to generate a high marker density map for chromosome 6 V, which will be of great importance for wheat molecular breeding and gene cloning [22].

## Materials and Methods

### Seed Material

A large number of seeds of the *D. villosum* DV-200 accession collected from Bomarzo (Viterbo, Italy) was kindly provided by Ciro De Pace (University of Tuscia, Viterbo, Italy). A set of disomic addition lines of *D. villosum* in common wheat cv. Chinese Spring (CS), each containing one pair of the V genome chromosomes of known homeology to wheat, were produced by E.R. Sears and A.J. Lukaszewski at the University of Missouri, Columbia, MO, and provided for this study by A. J. Lukaszewski, University of California, Riverside, CA, together with three different accessions of *D. villosum* used for the set production, called ‘Greek’, ‘Italian’ and ‘Sicilian’ and maintained by E.R. Sears (A.J. Lukaszewski, personal communication).

### Cell Cycle Synchronization

Cell cycle synchronization and accumulation of mitotic metaphase chromosomes was accomplished according to Vrana *et al.* [25] with minor modifications. Briefly, for each experiment a lot of 100 seeds was soaked in aerated water for 24 h and germinated on moist filter paper for 2 days at 25°±0.5°C in the dark. Young seedlings were transferred in an aerated Hoagland’s solution [26], at 25°±0.5°C in the dark. After an acclimatization period of 4 h, cell cycle synchrony was induced by incubation in 2 mM hydroxyurea (HU) for 18 h, followed by 4 h recovery period and an accumulation of cycling cells in metaphase by a treatment with 2,5 µM APM (amiprophos-methyl) for 2.5 h, followed by an overnight incubation in ice water.

### Chromosome Suspension

Chromosome suspensions were prepared following previous protocols [25] [27]. Cut root-tips were fixed in 2,5% (v/v) formaldehyde in Tris-HCl buffer solution pH 7.5 [28] at 4°C for 30 min. After three washes in Tris-HCl buffer pH 7.5, 1 mm root tips were cut and transferred to 1 mL of the LB01 buffer [29] at pH 7.5. Mechanical disruption was for 9 s at 9500 rpm in the ULTRA-TURRAX T8 with G5 generator (IKA, Staufen, Germany) in polystyrene tubes (FALCON 2054). The suspension was filtered through a 36 µm pore-size nylon mesh to remove tissue fragments and other debris, and about 5×10^5^ mL^−1^ intact chromosomes were isolated. Chromosome suspensions were stained with DAPI (4,6-diamidino-2-phenylindole) at the final concentration of 2 µg mL^−1^.

### Chromosome Analysis and Sorting

Chromosome samples were analysed using a FACS Vantage SE flow cytometer (Becton Dickinson, San Josè, CA) equipped with an argon ion laser Innova Coherent 90/5UV, with emission at **λ** = 353+361 nm and 200 mW power output for DAPI excitation. Fluorescence (FL1) was collected through a band-pass specific filter at **λ** = 420/30 nm. The Vantage SE was equipped with a 70 µm flow tip running at 27 psi using a solution of 50 mM NaCl as the sheath fluid. 20,000 particles were analyzed from each sample, and data were collected and analyzed using the CellQuest Pro 4.01 software (Becton Dickinson, La Jolla, CA). Sorting was performed at the 29.7 kHz drop drive frequency at the sorting rate of 5÷20 s^−1^ in the dual sorting mode, and chromosomes were collected onto a glass slide for immediate identification. The results were displayed as histograms of relative fluorescence intensity (flow karyotype). Sorting windows were drawn on a fluorescence dot plot of FL1 Area (DAPI, DNA fluorescence) versus Forward Scatter Height (FSC, light diffraction pattern). In order to verify chromosome content of individual peaks on the flow karyotype, 1000 chromosomes were sorted from each sorting region onto microscope slides into 10 µL of the LB01 buffer plus 5% sucrose [30] and air dried. The identity of the flow-sorted chromosomes was determined by microscopic observation after FISH labeling with the (GAA)_7_ probe. The purity of sorted fractions was assessed according to Vrana *et al.* [25] after identification of 100 labeled chromosomes per slide, counting three slides for sorting gate.

### Preparation of Mitotic Metaphase Spreads

In order to characterize single *D. villosum* chromosomes and to define their GAA karyotype, FISH was carried out on mitotic metaphase spreads prepared from root tips as described by Schwarzacher and Heslop-Harrison [31]. Briefly, after cell cycle synchronization, root tips were fixed in 3∶1 ethanol-glacial acetic acid overnight. They were then enzimatically digested and squashed in a drop of 45% acetic acid. The coverslips were removed by freezing and the slides were air dried for metaphase index (MI) evaluation and/or FISH labeling.

### Fluorescence in situ Hybridization (FISH)

Two DNA clones pSc119.2, (derived from a rye highly repeated sequences [32]) and pHv62 (genome V specific repeated sequence cloned from *D. villosum* [33]) were directly labeled by polymerase chain reaction with FITC (fluorescein-5-isothiocyanate) or Cy3 (Cyanine 3) using universal M13 primers and the corresponding DNA clones as templates. The 18S-5.8S-26S rDNA clone pTa71 [17] was labeled with Cy3 by nick-translation using standard kits (Nick Translation Mix, Roche) following manufacturer’s instructions. The high-performance liquid chromatography-desalted synthetic oligonucleotide (GAA)_7_ was purchased at Eurofins MWG Operon (Ebersberg, Germany), as single (Cy3) or double (FITC) terminal modifications, and suspended at 1 µg µl^−1^ final concentration in 10 mM Tris-HCl, 1 mM EDTA, pH 8.0.

Pretreatment and stringency washes [31] were applied only to the slides hybridized with pTa71, pSc119.2 and pHv62 probes. These steps were omitted in experiments with the GAA probe on root tip metaphase cells and on flow sorted chromosomes. FISH with pSc119.2, pHv62 and pTa71 was performed on metaphase spreads according to Schwarzacher and Heslop-Harrison [31]. The hybridization mixture (50 µL) containing (final concentration) 50% formamide, 2×SSC (0.30M NaCl, 0.030 M sodium citrate), 10% dextran sulfate, 30 ng µL^−1^ denatured salmon sperm DNA, 100 ng probe and water to make up the final volume, was spread on slides and sample DNA was denatured at 80°C for 5 min in a thermalcycler. Hybridization was overnight at 37°C in a moist chamber. The most stringent washes were performed twice in 20% formamide in 0.1×SSC at 42°C for 5 min. After DAPI (0.2 µg mL^−1^) DNA counterstaining, the slides were mounted in Vectashield antifade solution (Vector Laboratories, Burlingame, CA).

A fast labeling by FISH in non denaturing conditions (ND-FISH) was performed on metaphase spreads according to Cuadrado *et al.* [34], with minor modifications. Briefly, 50 ng of the (GAA)_7_ probe suspended in 30 µL of 2×SSC was added to the samples, and the slides were incubated at room temperature for 1 h; washed for 10 min in 4×SSC with 0.2% Tween20, counterstained with DAPI and mounted in Vectashield.

FISH on sorted chromosomes was carried out according to Kubalakova *et al.* [35] using the (GAA)_7_ oligonucleotide as a probe. The hybridization mix, consisting of 40% w/v formamide, 20% w/v dextran sulfate, 1×SSC, 250 ng µL^−1^ sheared calf thymus DNA and 80 ng labeled probe/slide (final concentration), was denatured for 10 min at 80°C, applied to the sample, and the slide was enclosed with a plastic coverslip. DNA was denatured on a thermalcycler for 40 s at 80°C, and the hybridization was at 37°C overnight. A stringent wash was performed in 0.1×SSC, 2 mM MgCl_2_ and 0.1% v/v Triton X-100 for 10 min at 42°C. Chromosome preparations were counterstained with DAPI 0.2 µg mL^−1^ and mounted in Vectashield.

### Microscopic Analyses

FISH-labeled chromosomes were analyzed under a Nikon Eclipse TE2000-S inverted microscope equipped with an HB0 100 W lamp and a PlanApo oil objective 100X and appropriate filter sets for DAPI, fluorescein and Cy3 fluorescence. Separate images from each filter set were captured and digitalized using a DXM1200F camera and the NIS AR 3.1 software (Nikon Instruments S.p.A, Florence, Italy). The images were superimposed on one another, after contrast and background optimization, using the ImageJ v1.46 free software (rsbweb.nih.gov/ij/index.html), and the *D. villosum* idiogram was generated using the ImageJ plugin CHIAS IV [36]. Chromosome morphology was measured by using the ImageJ free plugin LEVAN (http://rsbweb.nih.gov/ij/plugins/levan/levan.html) and the statistical significance of the differences among chromosome arm ratio were analysed using the Scott-Knott test [37] with the free Excel plugin DSAASTAT vers. 1.1 (http://www.unipg.it/~onofri/DSAASTAT/DSAASTAT.htm).

## Results

### The (GAA)_7_ FISH Karyotype

**Figure 1:**
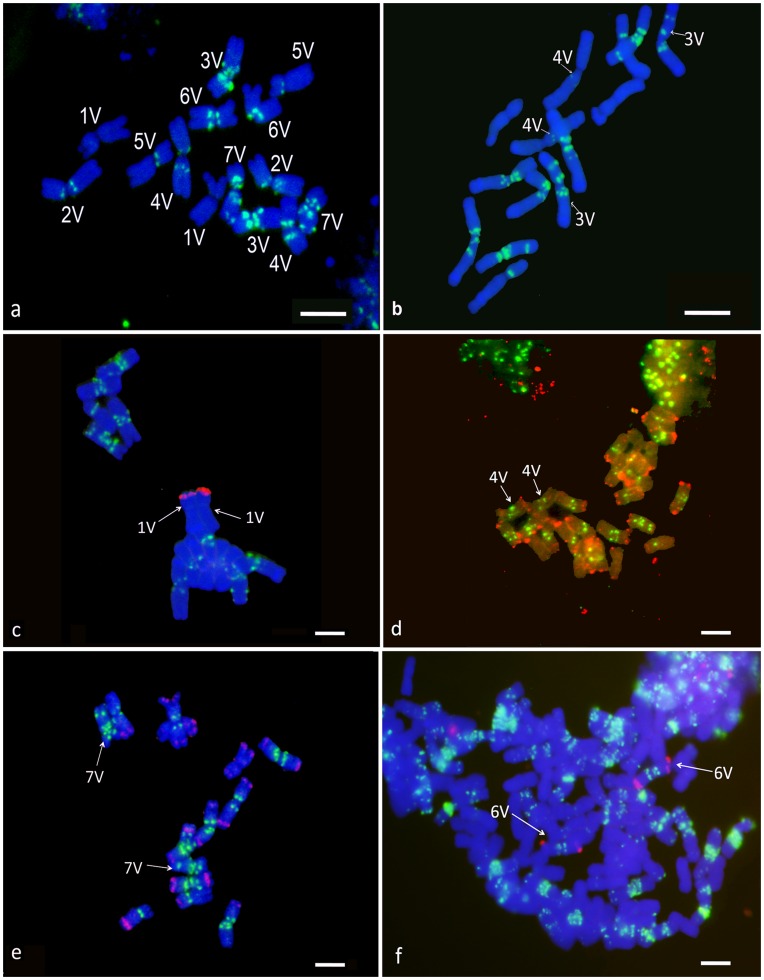
*D. villosum* chromosome identification by FISH labelling. Specific chromosomes and fluorescent bands are indicated by numbers and arrows, respectively. Metaphase chromosomes of: (a) *D. villosum* Bomarzo accession and (b) *D. villosum* ‘Greek’ accession, both after ND-FISH (GAA)_7_−FITC labeling (*green fluorescence*) and DAPI staining (*blue fluorescence*) are shown. All *D. villosum* Bomarzo chromosomes display a characterizing banding pattern while *D. villosum* ‘Greek’ accession chromosomes 3 V and 4 V differ in the GAA hybridization pattern, with respect to the Bomarzo accession. (c) Identification of chromosome 1 V in metaphase chromosomes of *D. villosum* Bomarzo after double target FISH with pTa71-Cy3 (*red fluorescence*) and (GAA)_7_−FITC. (d) Identification of chromosomes 4 V with double target FISH labeling with pSc119.2-Cy3 and (GAA)_7_−FITC oligonucleotide to *D. villosum* Bomarzo metaphase spreads. The pSc119.2 probe hybridized to the 4VS arm only. (e) Metaphase chromosomes of *D. villosum* Bomarzo after double FISH with pHv62-Cy3 and (GAA)_7_−FITC oligonucleotide; chromosome 7 V has been identified by pHv62 labeling. (f) Metaphase chromosomes of *T. aestivum* CS-6V *D. villosum* addition line after double FISH labeling of pHv62-Cy3 and (GAA)_7_-FITC oligonucleotide. The *D. villosum* 6 V chromosome added to *T. aestivum* CS standard complement has been identified by pHv62, a V genome specific probe.

*In situ* probing with the (GAA)_7_ oligonucleotide produced several clear and strong hybridization signals, mainly localized at the centromeric and pericentromeric regions on all chromosome pairs of *D. villosum* but one (Figure 1; Figure S1 and S2).

**Figure 2:**
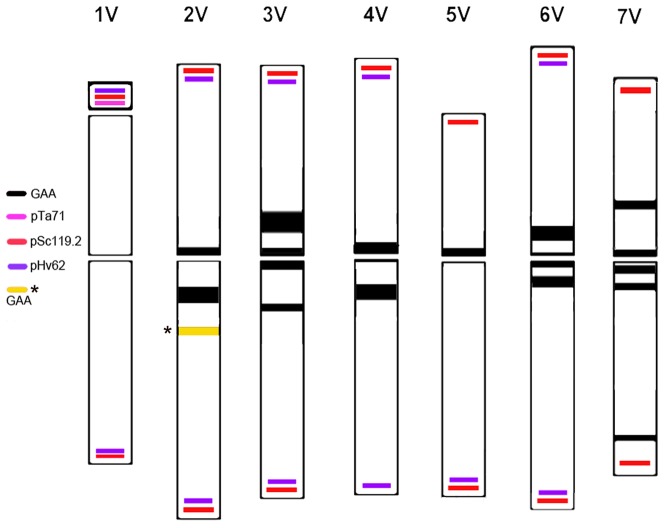
Ideogram of *D. villosum* chromosomes with the FISH probes labeling patterns. Ideogram showing the chromosomal distribution of the (GAA)_7_ oligonucleotide and of the three repetitive DNA sequences pTa71 (pink bar), pSc119.2 (scarlet bar) and pHv62 (violet bar). GAA bands are shown in black, but the extra yellow band with a star which is detectable only on sorted chromosome 2 V and it is lacking on metaphase spreads. The size of the GAA bands is arbitrarely related to the intensity of the hybridization signals.

The identification of *D. villosum* chromosomes and localization of the signal corresponding to their specific (GAA)_7_ hybridization pattern was performed by double target FISH experiments on metaphase spreads using the (GAA)_7_ oligonucleotide in combination with one of the following probes: pTa71, pSc119.2, pHv62. Such probes have been previously used by Uslu *et al.* [15] to characterize individual chromosomes of *D. villosum* in disomic *Triticum aestivum* addition lines, thus enabling us to unambiguously identify chromosome pairs 1 V, 4 V and 7 V. In particular, probe pTa71, containing 18S-5.8S-28S rDNA, allowed the discrimination of the chromosome pair 1 V (Figure 1c), the only chromosome where such genes are known to be located [15]. Probe pSc119.2 enabled the identification of chromosome 4 V, as it hybridizes only to the telomere of the short arm on this chromosome but to both telomeres of all the other chromosomes (Figure 1d and Figure 2). Finally, chromosome 7 V has been identified by the absence of a hybridization signal by the pHv62 probe, which showed fluorescent bands on all the other *D. villosum* chromosomes (Figure 1e). The chromosome pairs 2 V, 3 V, 5 V and 6 V were identified using the *Triticum* aestivum CS-*D. villosum* addition lines hybridized with the (GAA)_7_ probe. As this probe also efficiently labels wheat chromosomes, we used it in a double target FISH joined with the V genome-specific probe pHv62 to discriminate each of the four *D. villosum* chromosomes from the CS complement. As an example of double FISH labeling, a metaphase of a CS-6V *D. villosum* addition line is shown in Figure 1f.

The (GAA)_7_ hybridization pattern analysis enabled the unambiguous discrimination of five out of the seven chromosome pairs of the *D. villosum* complement with the only exception of the pairs 2 V and 4 V. In Figure 1 and Figure S1, *D. villosum* chromosome pairs identification is presented: chromosome pair 1 V got no GAA hybridization signals (Figure 1, a and c), chromosome pair 5 V displayed a strong signal at the centromeric region only (Figure 1a); chromosome pair 7 V showed a distinctive hybridization site at the telomeric region of 7VL, together with three bands at the pericentromeric and centromeric regions of the long and short arms (Figure 1, a and e). Chromosome pairs 3 V and 6 V showed three hybridization signals localized at the centromere and at the pericentromeric regions of both arms, but on chromosome 3 V all the centromeric and pericentromeric regions appeared strongly GAA labeled and the signal intensity was brighter than on the 6 V pair. On the 2 V chromosome the (GAA)_7_ produced two bright signals: one located at the centromere region and the second one at the near pericentromeric region of the 2 VL arm (Figure 1a; Figure S1). A similar pattern was detected on the metacentric chromosome 4 V and a morphological analysis proved to be of use to discriminate each of the two chomosomes. The whole complement has been measured for chromosomes short and long arm length on twelve good metaphase spreads (Figure S1) and the chromosomes with similar arm ratio were clustered by the Scott-Knott test [37] (Figure S2). Chromosomes 4 V and 6 V showed up as metacentrics (group**^b^**); four chromosomes were cataloged as near metacentric (1 V, 2 V, 3 V and 7 V; group**^a^**) with the 5 V chromosome classified as a submetacentric (group**^c^**), so discriminating the 2 V and 4 V pairs in two different morphological groups (Figure S2). When sub-optimal metaphase spreads are available, the morphological differences among the two chromosome pairs are difficult to be detected and the chromosome 4 V could be discriminated from the 2 V one by double labeling with the probes GAA and pSc119.2, the latter labels 4VS only but both arms on 2 V (Figure 1 c and Figure 2). We could not discount the possibility that the karyotype of the *D. villosum* accession “Bomarzo” used in this study differed from the reference karyotype described by Uslu *et al.* [15], thus we performed a number of preliminary FISH analyses, using a set of probes (pTa71, pSc119.2, pHv62 and (GAA)_7_) on mitotic metaphase spreads of the *D. villosum* Bomarzo accession and the three accessions, ‘Greek’, ‘Italian’ and ‘Sicilian’ used in the development of CS-*D. villosum* addition lines [15]. No major differences in signal distribution were observed for any probes among the three accessions, thus confirming the utility of such repetitive sequences for the identification of individual chromosomes of *D. villosum*. On the contrary, some minor differences in the GAA distribution were detected among the three listed accessions and the Bomarzo one; specifically, a subtelomeric hybridization signal was present on chromosome 3VL in the ‘Greek’, ‘Italian’ and ‘Sicilian’ accessions, but absent on the Bomarzo one. In addition, a centromeric signal was detected on Bomarzo chromosome 4VS, no evident in the other accessions (Figure 1, a and b). However, chromosome discrimination was not hindered by these differences. The non random distribution of the GAA repetitive sequences on *D. villosum* chromosomes is summarized in Figure 2, where it is presented the first corresponding ideogram, with all the labeling probes located on each chromosomes.

### Chromosome Suspensions and Flow Sorting

**Figure 3:**
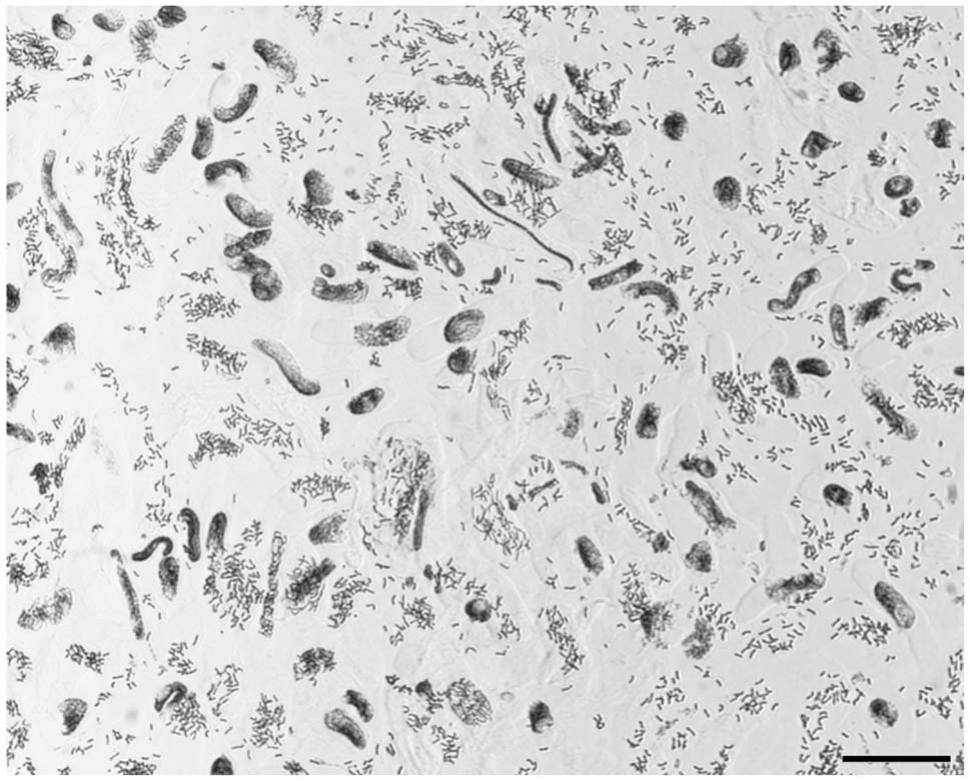
*Dasypyrum villosum* metaphases after a “dual blocking steps” cell cycle synchronization procedure. A high metaphase index (>60%) has been achieved in *D. villosum* root tips after cell cycle synchronization and metaphase enrichment was induced by incubation in 2 mM hydroxyurea for 18 h, followed by 4 h recovery and a metaphase block after a treatment with 2,5 µM amiprophos-methyl for 2,5 h, followed by overnight incubation in ice water. Feulgen-stained chromosomes are evenly distributed all over the observation field. Scale bar = 100 µm.

**Figure 4:**
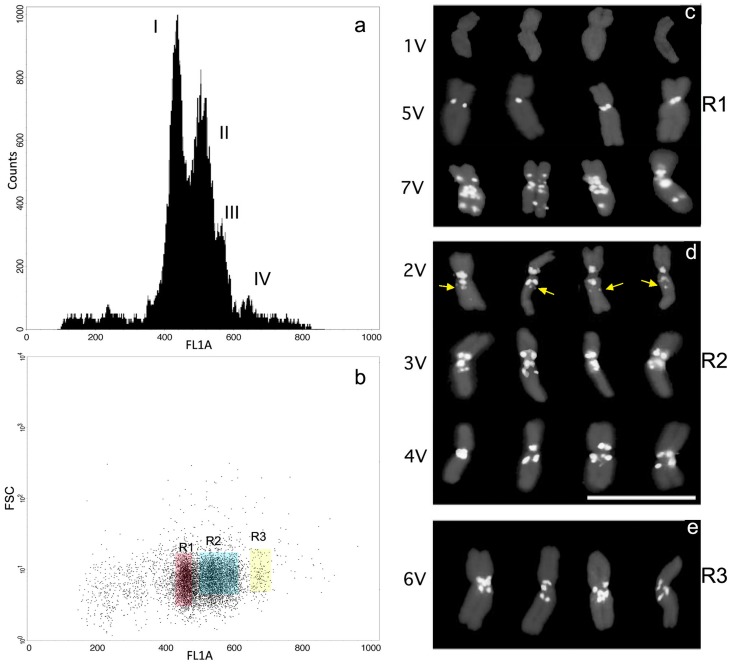
*Dasypyrum villosum* FCM karyotyping and chromosome identification after flow sorting. (a) The flow karyotyping obtained after analysis of DAPI-stained chromosomes show three composite peak (I, II and III) and one peak apart (IV). (b) Sorting regions are drawn around chromosome distribution areas as defined on a fluorescence dot plot of the FL1A (FL1 area, DNA, fluorescence) versus the FSC (chromosome forward scatter). Only three regions, named R1, R2 and R3, were located since the main chromosome distribution did not allowed to focus single chromosomes except a single peak, named region R3. All sorted chromosomes were identify by FISH labelling with (GAA)_7_−Cy3 and DAPI staining. In (c-e) images of flow sorted chromosomes from each region are reported. R3 contained a single chromosome (e), identified as 6 V. In R2, an additional GAA band is shown on chromosome 2 V (yellow arrow), which is not present on 2 V metaphase chromosomes and facilitate its discrimination from the 4 V ones in sorted fractions. Scale bar = 10 µm.

High quality suspensions of *D. villosum* chromosomes were obtained following the isolation protocol described by Vrana *et al.* [25] with some modifications to improve chromosome quality and the flow karyotype resolution. After double cell cycle synchronization with HU and APM, resulting in a MI of 60% (Figure 3), best *D. villosum* karyotype resolution (Figure 4a) was obtained increasing formaldehyde fixation up to 30 min at a homogenization speed at 9.500 rpm for 9 s.

*D. villosum* chromosomes in suspensions were stained with DAPI and analysed by flow cytometry to originate a DNA content fluorescence intensity histogram showing four peaks (Figure 4a), where only the fourth peak on the right was unambiguously identified and containing a chromosome with the higher DNA content, mainly 6 V (Figure 4e). The other three peaks were difficult to discriminate each other since the remaining chromosomes exhibit a similar size, and therefore a near fluorescence distribution (Figure 2 and Figure S2).

Sorting regions were drawn on a dot plot in which the DNA peaks fitted into three regions because of the close correspondence of the chromosome DNA fluorescence signals which raised a substantial overlapping fluorescence intensity emission preventing peak II to be neatly resolved from peak III (Figure 4b). The chromosome content of each region was determined by FISH on flow-sorted chromosomes using the (GAA)_7_ probe. The sorting region R1 on the dot plot (Figure 4b), corresponding to peak I (Figure 4a), contained an equal distribution of chromosomes 1 V, 5 V and 7 V (Figure 4c); the region R2 (Figure 4b) clustered peak II and peak III and comprised a mixture of chromosomes 2 V, 3 V and 4 V (Figure 4d); the region R3 (Figure 4b), corresponding to peak IV, contained a single chromosome type, namely 6 V (Figure 4e) which could be sorted with a purity >85% as microscopically determined after (GAA)_7_ labeling.

We have also found that a lesser chomosome condensation, such as of flow sorted chromosomes, helps to get a better discrimination on bands number, location and intensity, as is the case for an extra GAA band which showed up on the sorted chromosome 2 VL (Figure 2 and Figure 4d), allowing an easier discrimination among 2 V and 4 V.

## Discussion

*D. villosum* is an important species for wheat breeding and carries many genes of interest such as loci for biotic and abiotic stress resistance and for storage proteins that affect the amount, yield and quality of seed proteins [7], [8]. Over the years, efforts have been focused on the introduction of useful genes from *Dasypyrum* into *Triticum* genome backgrounds by addition, substitution and translocation lines [11], [38], [39]. FISH with repetitive DNA sequences and GISH (genomic *in situ* hybridization) have been widely used both to characterize *D. villosum* chromosomes [15] and to analyze aneuploid lines and interspecific hybrids genomes [15], [16], [40].

Repetitive DNA sequences such as satellite DNA, simple sequence repeats (SSRs) and transposable elements are spread into plant genomes [41], [42], [43] both organized in a tandem fashion, to form large blocks (mainly satellite families, SSR and same gene families, e.g. ribosomal genes) or dispersed throughout the entire genome (transposable and retrovirus-related elements). Several *Triticeae* species have been studied for their genome organization and had individual chromosome identified by tandem repeated sequences [44], [45], [46], [47], [48], [49], [50]. In particular, the GAA SSR microsatellite sequence has been extensively used to explore *Triticeae* genomes and to identify chromosomes in several grain species [27], [51], [52], [53]. Pedersen *et al.* [53] reported a high frequency of the GAA repeat in the *D. villosum* genome, assessed by the Southern blot hybridization, but to our knowledge no GAA FISH karyotyping has been done in *D. villosum* so far. Here we present the first GAA karyotype of *D. villosum* (Figure 1a; Figure S1) providing a clear evidence that the (GAA)_7_ hybridization pattern on metaphase spreads, allowed the unambiguous discrimination of chromosomes 1 V, 3 V, 5 V, 6 V and 7 V. The remaining chromosome pairs 2 V and 4 V exhibited a similar GAA banding but could be discriminated by the observation of the chromosomes primary constriction position and their arm ratio analysis. Statistically significant differences among the arm ratios of the two chromosomes have been clustered in different groups [37], thus enforcing the true discrimination of *D. villosum* chromosomes by a joint FISH labeling and morphological approach. When chromosomes are highly condensed and the morphological differences are not easy detectable on microscope, a double target FISH labeling with the pSc119.2 and (GAA)_7_ probes can make the identification of chromosomes 4 V feasible (Figure 1d; Figure 2). The GAA FISH pattern in *D. villosum* does not resemble the pattern of C-bands [16], [54], as is the case in wheat and in rye [53]. Specifically, thick blocks of telomeric and subtelomeric heterochromatin revealed by C-banding are clearly not composed of the GAA repeat and do not show up in FISH. Simultaneous hybridization with the (GAA)_7_ oligonucleotide and the V genome-specific pHv62 probe produces a labeling pattern very similar to C-bands (Figure 2). Moreover the combination of such probes in double target FISH to the CS-*D. villosum* addition and substitution lines makes possible an unambiguous identification of the *D. villosum* chromosomes in the CS background (e.g. Figure 1f).

A minor polymorphism in the numbers of the GAA hybridization sites was detected between *D. villosum* Bomarzo and the three parental accessions used to make the CS-*D. villosum* addition lines (Figure 1, a and b). Intraspecific polymorphism, both in the number and in the distribution of repeated DNA sequences, has been reported in numerous species, including different cultivars of wheats [35], but in most cases, chromosomes identification is not hindered by such polymorphism [55]. Our observations on the low level of the GAA FISH pattern polymorphism among the *D. villosum* population collected in Bomarzo, with respect to the three accessions previously collected in Greece, Central Italy and Sicily, could be explained by geographic isolation in which the Bomarzo accession grows, which in turn could account for genetic drift occurring during its evolution [7].

On the last few years, the FC approach has been used to dissect complex plant genomes of high economic relevance, (i.e. wheat, barley, rye), with the aim of allowing genomic studies and genome sequencing [56]. “Chromosome genomics” [23] facilitates the analysis of molecular structure of chromosomes, the development of molecular markers, the construction of physical maps and positional cloning. The usefulness of the FC approach is demonstrated by a number of relevant applications that rely on sorted chromosomes [57]. Wenzl *et al.* [22] have demonstrated that the DArT (Diversity ARrays Technology) chromosome-specific markers can be developed in large numbers directly from small amounts of DNA obtained from flow-sorted chromosomes. Besides, the advent of the next-generation sequencing technologies, allowing the study of the molecular composition of individual chromosomes, may provide almost unlimited numbers of sequences suitable for the development of molecular markers, such as SSR and single nucleotide polymorphism, of high value for marker assisted selection.

Recently, the potential of flow cytogentics has been extended to wild relatives of wheat belonging to the genus *Aegilops* [24]. Here, we further expand flow cytogenetics to another wild species, *D. villosum,* describing its flow karyotype and demonstrating that the diploid genome can be fractioned into three sections, one of which contains a single chromosome (Figure 4). From our previous experience in chromosome isolation [58], we understand that the main issues in optimizing chromosome isolation in suspension are: (i) a high MI after cell cycle synchronization (Figure 3); (ii) the fixation time with formaldehyde; and (iii) the speed and the time duration of the tissue mechanical disruption. Careful set-up of those experimental factors allowed the isolation of numerous and intact *D. villosum* chromosomes in suspension, suitable for flow karyotyping. As flow cytometry analysis of chromosomes is based on their relative intensity of fluorescence after staining with a DNA-specific dye (DAPI), it follows that the flow karyotyping chromosome distribution is directly correlated with chromosome size. The flow karyotype of a natural *D. villosum* population consists of four peaks containing all seven chromosomes of the species (Figure 4a). Because of their similar dimensions, and therefore near DNA content of all the *D. villosum* chromosomes (Figure 2, Figure S2), the distribution of the intensity of the chromosome DNA fluorescence signals did not generate four well separated peaks, which in turn produced sorting regions including a number of different chromosome types. At best of flow karyotyping in a real world, a complete separation of chromosome peaks requires a DNA content difference of 6–10% among analyzed chromosomes, most depending on the amount of cellular debris occurring during the chromosome isolation procedure [57]. The GAA FISH analysis of the flow sorted fractions showed that only region 3, including peak IV, contained a single type of chromosome, namely 6 V, at a purity above 85%. The identification of the chromosome content of the flow karyotype peaks and the purity of the sorted fractions were best evaluated by microscopic assessment of their morphology and FISH labeling with the GAA probe (Figure 4). Using this approach, we were able to identify the effective sorting regions R1, R2 and R3 and to characterize their chromosome content type. This methodology proved to be superior to molecular approaches as it allows determination of the frequency as well as the nature of all contaminating particles [59]. Moreover, FISH on sorted chromosomes enabled better hybridization signal resolution compared with FISH on metaphase spreads, probably due to a lesser condensation of chromosomes and to the absence of cytoplasm and cellular debris [60]; this was the case for chromosome 2 V, where an additional GAA band showed up on the 2 VL arm of the sorted chromosome (Figure 4d), allowing an easier identification of chromosome 2 V. Combining all the information from the probes (GAA)_7_, pTa71, pSc119.2 and pHv62 FISH banding patterns on metaphase spreads and the chromosome measurements, we characterize and drew the first ideogram of the whole complement of *D. villosum* (Figure 2; Figure S2). The availability of its chromosomes in suspension permits flow karyotyping and flow sorting which makes accessible further cytogenetic infomations (Figure 4d), thus allowing the identification of all the *D. villosum* chromosomes by the (GAA)_7_ probe and their morphology only.

The successful flow sorting of chromosome 6 V of *D. villosum* will provide a valuable genetic resource because it is known to carry several valuable genes for resistance and/or tolerance to biotic and abiotic stresses and for seed storage protein traits [11], [61], [62], [63], [64], [65]. The possible exploitation and isolation of such useful traits relies on the availability of molecular tools, including DNA markers, of which there are very few and they are dispersed on several chromosomes [8]. The chance to generate pure samples of the target chromosome 6 V creates opportunities for the development of large numbers of new and specific molecular markers. In turn, this will permit the construction of a dense genetic map, useful for gene mapping and molecular breeding. In our opinion, the high discrimination power of the GAA FISH in *D. villosum* may eventually allow flow sorting of all seven chromosomes of the complement on the basis of the GAA fluorescence patterns, if an effective method for FISH on chromosomes in suspension could be developed.

## Supporting Information

Figure S1 ***D. villosum*** **metaphase spreads.** Metaphases of *D. villosum* Bomarzo accession after ND-FISH with a (GAA)_7_− FITC oligonucleotide probe and DAPI staining. All the seven chromosome pairs are identified looking at their hybridization pattern and centromere position. Scale bar = 10 µm.
(TIF)

Figure S2 ***D.villosum*** **chromosomes characterization with GAA banding and morphological parameters.** A cluster analysis of the chromosome arm ratio (long/short arm) using the Scott-Knott test [37] showed not significant differences (P = 0.05) among values labeled with the same letters. Three groups of similarity are defined which allow the identification of all the *D. villosum* chromosomes in combination with the GAA FISH labeling pattern. The chromosome length is given as a percentage of the total chromosomre complement span, with the corresponding sorting region specified.
(TIF)
