# Supplementary figures and images for: Flow Sorting and Molecular Cytogenetic Identification of Individual Chromosomes of Dasypyrum villosum L. (H. villosa) by a Single DNA Probe
Source: PLoS One. 2012 Nov 20;7(11):e50151. doi: 10.1371/journal.pone.0050151 (PMC3502404; doi:10.1371/journal.pone.0050151)

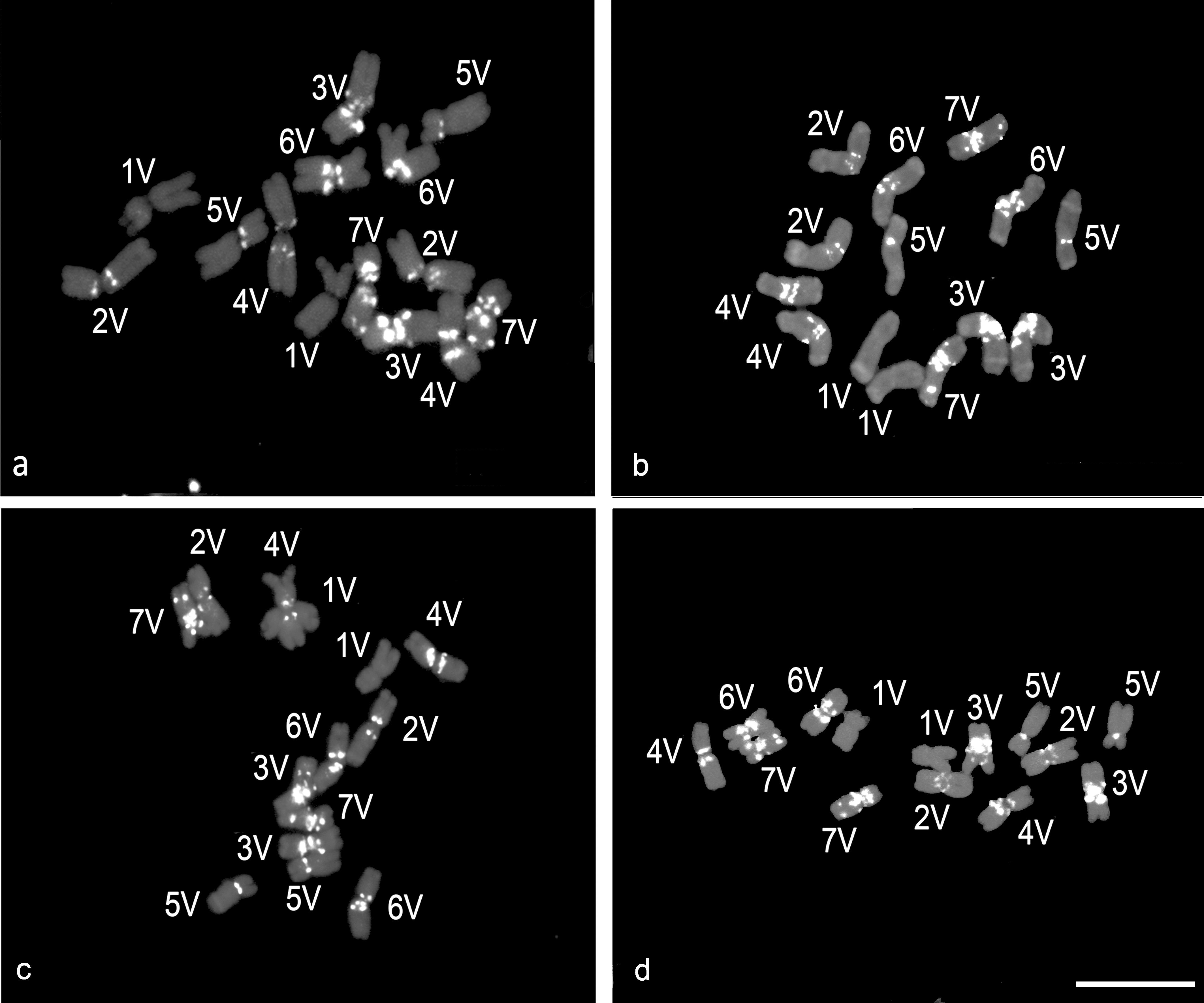

Supplement: Figure S1 — D. villosum metaphase spreads. Metaphases of D. villosum Bomarzo accession after ND-FISH with a (GAA)7− FITC oligonucleotide probe and DAPI staining. All the seven chromosome pairs are identified looking at their hybridization pattern and centromere position. Scale bar = 10 µm. (TIF) [file pone.0050151.s001.tif]

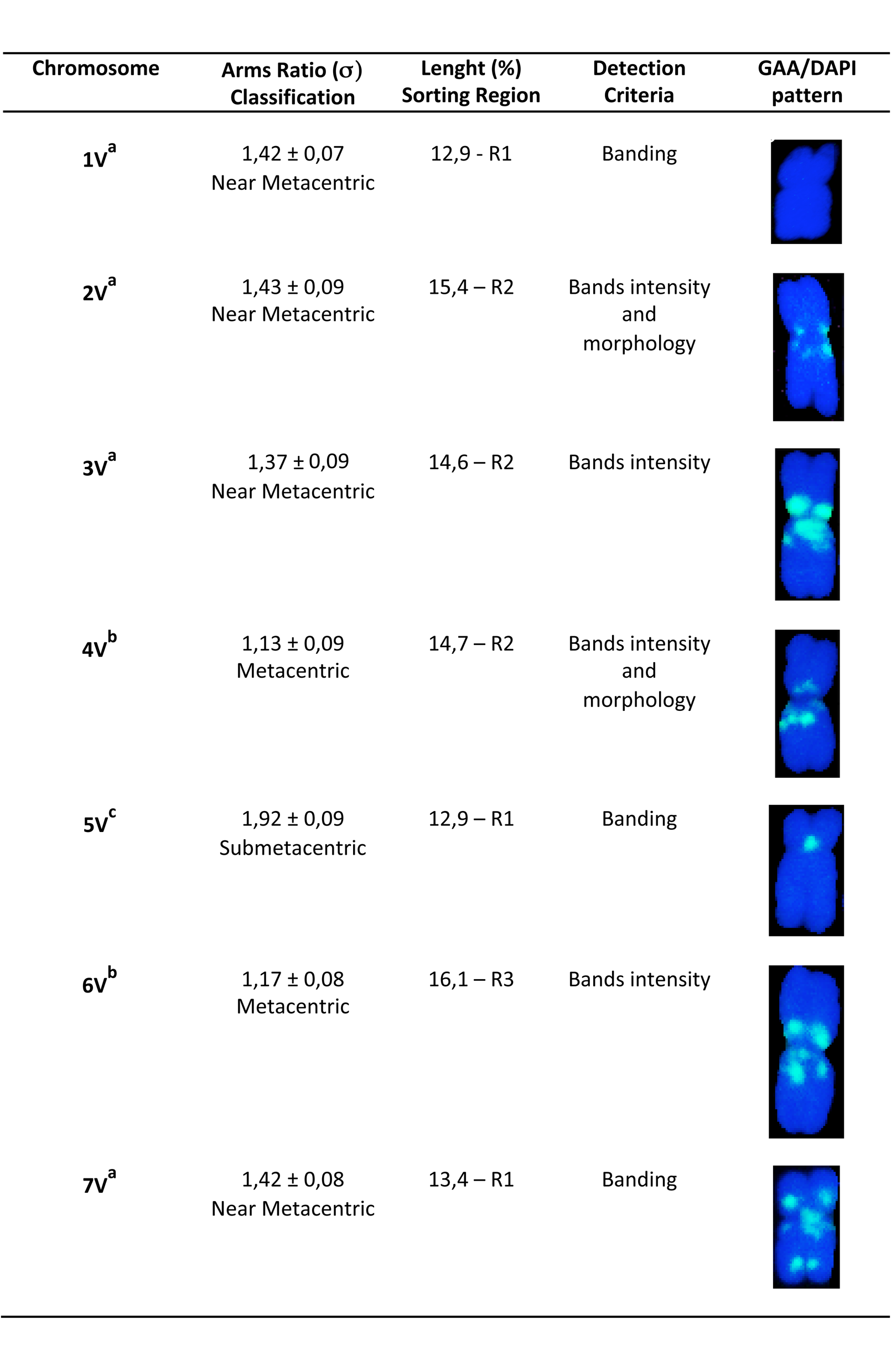

Supplement: Figure S2 — D.villosum chromosomes characterization with GAA banding and morphological parameters. A cluster analysis of the chromosome arm ratio (long/short arm) using the Scott-Knott test [37] showed not significant differences (P = 0.05) among values labeled with the same letters. Three groups of similarity are defined which allow the identification of all the D. villosum chromosomes in combination with the GAA FISH labeling pattern. The chromosome length is given as a percentage of the total chromosomre complement span, with the corresponding sorting region specified. (TIF) [file pone.0050151.s002.tif]
